# New Phylum Names Harmonize Prokaryotic Nomenclature

**DOI:** 10.1128/mbio.01479-22

**Published:** 2022-09-15

**Authors:** Aharon Oren, Markus Göker, Iain C. Sutcliffe

**Affiliations:** a The Hebrew University of Jerusalem, The Institute of Life Sciences, Edmond J. Safra Campus – Givat Ram, Jerusalem, Israel; b Leibniz Institute DSMZ – German Collection of Microorganisms and Cell Cultures, Braunschweig, Germany; c Northumbria University, Faculty of Health and Life Sciences, Newcastle Upon Tyne, United Kingdom; The Ohio State University

**Keywords:** nomenclature, *Bacteria*, *Archaea*, International Code of Nomenclature of Prokaryotes

## LETTER

We are pleased that Panda et al. (1) applaud the work of the International Committee on Systematics of Prokaryotes (ICSP) in bringing consistency to phylum names by voting in 2021 to include the rank of phylum in the International Code of Nomenclature of Prokaryotes (ICNP) (2). We here respond to the alleged pitfalls of the proposed nomenclatural system for naming phyla under the rules of the ICNP (3).

Most validly published phylum names (4) differ from the earlier ones that lack standing in nomenclature. This does not contravene Principle 1 of the ICNP, which only applies to validly published names (Principle 7). Panda et al. (1) describe ICSP’s argument that name changes may only be problematic in the short term as “an entirely flippant viewpoint (p. 2).” We refute this interpretation: our response (5) to the paper by Lloyd and Tahon (6) was a serious and pragmatic assessment of the situation given the comparatively short time most colloquial names have been in use versus the “long future” that awaits the correctly formed phylum names.

Panda et al. (1) contend that the ICSP position that (“the community still decides which [names] to adopt”) is an “ambiguity … [that] … will add more confusion, as there is no uniform way to proceed (p. 2).” We instead suggest that the recent changes to the Rules of the ICNP now (for the first time) provide a “uniform way” to name phyla (2, 7) and hope that the wider community recognizes this by choosing to use the validly published (correct) names.

Panda et al. (1) wondered why phyla were named *Pseudomonadota* and *Aquificota*, and not *Pseudomonasota* and *Aquifexota*, and claimed that suffixes have been haphazardly appended to genus names to name phyla. The proposal of these names (4) was not an action of the ICSP, but the relevant rules of the ICNP (2) were followed correctly: to name higher taxa, the appropriate suffix is added to the stem of the name of the type genus (Rule 9). The stem of Greek or Latin nouns or adjectives is generally found in the genitive case (8, 9). Concerning the question whether “the foremost determined type genus” is “a reliable representative for its respective taxon” (p. 4): Rule 15 states that a nomenclatural type is not necessarily the most typical or representative element of the taxon. The type genus selected does not have to be the first described genus contained in the phylum, but authors are encouraged to respect priority by considering this (Rule 22). Panda et al. (1) suggested renaming some phyla and proposed names such as *Proteobacteriota* and *Firmicuteota*. However, such names contravene Rule 8 as they are not based on the name of a designated type genus.

The answer to the question by Panda et al. whether classes such as *Alphaproteobacteria* will be renamed *Alphapseudomonadota* is negative. Admittedly, there are inconsistencies in the naming of classes. However, the ICSP is already addressing retroactivity of Rule 8 to resolve the issue in its ongoing revision of the ICNP (10), and further proposals on how to deal with the current inconsistencies will be submitted by its editorial board for consideration by the ICSP after finalization of the 2022 revision of the ICNP.

Finally, Panda et al. (1) complained about lack of appropriate quality control for newly proposed names. Experts evaluate every new name of prokaryotic taxa submitted to the International Journal of Systematic and Evolutionary Microbiology (IJSEM). Publication of names that contravene the Rules of the ICNP seldom occurs. Validation of the name *Myxococcus llanfairpwllgwyngyllgogerychwyrndrobwllllantysiliogogogochensis* could not be denied, as Recommendation 6 (1) (“Avoid names or epithets that are very long or difficult to pronounce”) is not a Rule.

The ICSP encourages researchers to inform themselves in time about the rules of nomenclature (3) and about proposals to modify them (7, 11), to participate in ICSP debates (10), and to propose changes to the ICNP following the process defined in the ICSP statutes (12). For example, if Panda et al. believe archaeal phyla should have the suffix –*archaeota*, then a formal proposal to this effect could be made.
